# Dapagliflozin protects against dilated cardiomyopathy progression by targeting NLRP3 inflammasome activation

**DOI:** 10.1007/s00210-023-02409-5

**Published:** 2023-02-07

**Authors:** Jiaxin Hu, Jiamin Xu, Xi Tan, Dong Li, Dejiang Yao, Biao Xu, Yuhua Lei

**Affiliations:** 1 Cardiovascular Disease Center, The Central Hospital of Enshi Tujia and Miao Autonomous Prefecture, Enshi Clinical College of Wuhan University, No.158 Wuyang Avenue, Enshi, 445000 Hubei China; 2 Department of Cardiology, Nanjing Drum Tower Hospital, The Affiliated Hospital of Nanjing University Medical School, Nanjing, China; 3 Department of Cardiology, Nanjing Drum Tower Hospital Clinical College of Nanjing University of Chinese Medicine, Zhongshan Road, Nanjing, 210008 China; 4 Department of Medical Oncology, Enshi Tujia and Miao Autonomous Prefecture Central Hospital, Enshi, Hubei China; 5 Surgical Division IIIThe Central Hospital of Enshi Tujia and Miao Autonomous Prefecture, Enshi Clinical College of Wuhan University, Enshi, Hubei China

**Keywords:** Dilated cardiomyopathy, SGLT2 inhibitors, NLRP3 inflammasome, Inflammation, TLR4

## Abstract

Dilated cardiomyopathy (DCM) is the major cause of heart failure and has a poor prognosis. The accumulating evidence points to an essential role of the inflammatory component in the process of DCM. Inhibitors of sodium-glucose cotransporter 2 (SGLT2) are widely used to treat heart failure patients due to their cardiac benefits. However, their role in DCM remains unclear. We used the doxorubicin (Dox)-induced DCM model for our study. The SGLT2 inhibitor dapagliflozin (Dapa) improved cardiac function in mice treated with doxorubicin and attenuated the activation of the nucleotide-binding oligomerization domain-like receptor family protein 3 (NLRP3) inflammasome pathway and the expression of inflammatory factors. In addition, dapagliflozin suppresses NLRP3 activation by decreasing p38-dependent toll-like receptor 4 (TLR4) expression. In our study, dagliflozin improves cardiac function in DCM by inhibiting the activity of the NLRP3 inflammasome.

**Graphical Abstract:**

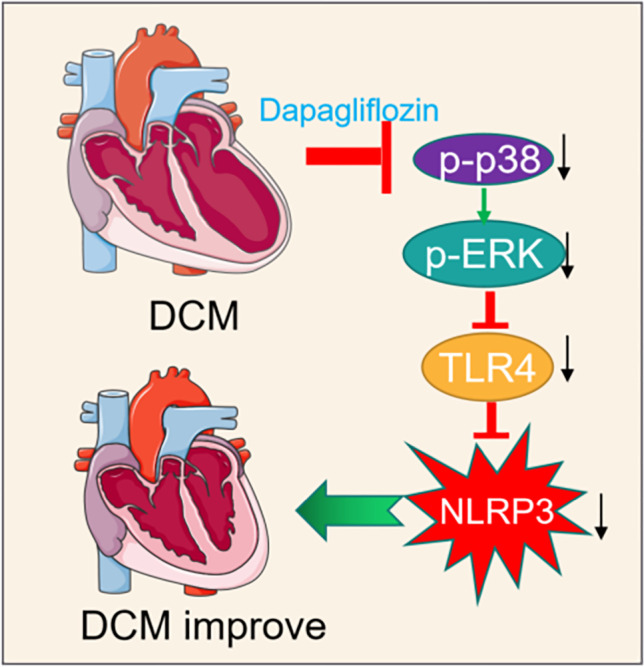

## Introduction

Dilated cardiomyopathy (DCM) is characterized by left ventricular dilation and systolic dysfunction, making it the most common cause of heart failure globally (Weintraub et al. 2017). It is defined by the WHO as a serious cardiac disease with severe morbidity and mortality due to complications such as heart failure and arrhythmias (Richardson et al. 1996). ​In epidemiological studies, DCM prevalence was estimated to be over one per 250 (Hershberger et al. 2013). In spite of improved treatment options in recent years, the prognosis for DCM remains poor, highlighting the importance of finding new treatments.

The pathogenesis of DCM is complex and primarily unclear, including genetic mutations, inflammation, infection, drugs and toxins, and autoimmune diseases (Schultheiss et al. 2019). ​The accumulating evidence points to an essential role of the inflammatory component in the process of DCM (Toldo et al. 2022). ​There is a strong link between caspase-1-dependent pyroptosis in cardiomyocytes and the nucleotide-binding oligomerization domain-like receptor family protein 3 (NLRP3) inflammasome (Zeng et al. 2020; Miao et al. 2010).​In DCM patients, circulating levels of NLRP3 inflammasome may be associated with cardiac function and rehospitalization (Luo et al. 2013). ​Considering DCM therapy’s inefficiency, targeting NLRP3 inflammasome activation and associated pyroptosis may be a potential therapeutic strategy.

Dapagliflozin, a sodium-glucose cotransporter 2 (SGLT2) inhibitor, is a common clinical drug for treating type 2 diabetes. ​Due to their additional cardiovascular benefits over and above glucose-lowering potential, SGLT2 inhibitors are now widely used in treating heart failure patients with or without diabetes (McDonagh et al. 2021). Furthermore, a recent study suggests that SGLT2 inhibitors may have anti-inflammatory effects by inhibiting NLRP3 inflammasome activation (Kim et al. 2020; Long et al. 2022). At the same time, the pyroptosis process is constrained due to suppressed caspase-1 activity and caspase-1-dependent release of the cytokines interleukin-1β (IL-1β) and IL-18 (El-Rous et al. 2021). Toll-like receptor 4 (TLR4) plays a vital role in the inflammatory response. In human tubular epithelial cells, SGLT2 inhibitors remarkably reversed glucose-induced TLR4 expression (Panchapakesan and Pollock 2021). Therefore, dapagliflozin’s effect on NLRP3 expression and TLR4 involvement in DCM should be investigated.

In the current study, we investigated the effects of dapagliflozin on the regulation of the NLRP3 inflammasome and protection against doxorubicin-induced cardiomyopathy. ​Here, we show that dapagliflozin significantly protects cardiac function during DCM progression via TLR4-mediated inhibition of NLRP3 inflammasome activation.​

## Methods

### Animal experiments

Male C57BL/6 mice (8–10 weeks of age) were purchased from the Model Animal Research Centre of Nanjing University. Mice were kept at Nanjing Drum Tower Hospital’s Animal Laboratory Resource Facility. All procedures using experimental animals were approved by Nanjing Drum Tower Hospital’s Institutional Ethics Committee, and the National Institutes of Health’s Guide for the Care and Use of Laboratory Animals (8th edition) was followed. All animals were euthanized by cervical dislocation after anesthetization with isoflurane (1.5–2%).

Twenty male C57BL/6 mice aged 8–10 weeks were given 5 mg/kg of doxorubicin intraperitoneally once a week for 4 weeks as the Dox group. Ten other male C57BL/6 mice were given equal amounts of PBS for 4 weeks as controls. The Dox group mice were randomly assigned to two groups; dapagliflozin (1.5 mg/kg/day) (Withaar et al. 2021) or saline was gavaged once daily for 4 weeks. After 4 weeks, the mice were sacrificed, and the left ventricular myocardium of the mice was harvested for correlation analysis.​

### Echocardiography

Four weeks after modeling, mice were anesthetized with 1–2% isoflurane for transthoracic echocardiography. By using M-mode tracings, left ventricular ejection fractions (LVEF), left ventricular fractional shortenings (LVEF), and left ventricular internal dimensions at end-diastole (LVIDd) and end-systole (LVIDs) were measured. The measurements were carried out by one experienced technician who was blinded to the study groups.

### Histological analysis and immunohistochemistry

Histological studies were conducted on mice after 4 weeks of modeling. We perfused the left ventricle with saline to remove the blood and then fixed the ventricular tissues in 10% formalin, transected, embedded in paraffin, and stained the 5 mm sections with hematoxylin and eosin (H&E) for morphological evaluation.

An immunohistochemistry study was conducted in order to detect the expression of interleukin (IL)-6 and IL-1β in the heart. The level of IL-6 and IL-1β was analyzed by measuring the optical density of the myocardium-stained area. Images were obtained using a conventional fluorescence microscope (Leica Thunder, Germany), and a blinded investigator counted positive regions.

### Immunofluorescent staining

Using citrate buffer, the myocardial tissue of mice was boiled for 1 h at 100 °C to obtain antigen, followed by blocking with 1% fetal bovine serum for 1 h at room temperature. The primary antibody was incubated overnight at 4 °C, followed by washing 2–3 times with PBS for 5 min each, followed by secondary antibody incubation for 1 h at room temperature. After staining with DAPI for 10 min at room temperature, the samples were washed twice with PBS under no light conditions. Confocal microscopy was used to photograph the samples immediately after blocking the anti-fluorescence quencher agent.

### Real-time polymerase chain reaction analysis

The total mRNA was extracted from heart tissue using RNAiso plus (Vazyme Biotech Co. Ltd). Subsequently, a real-time polymerase chain reaction (PCR) was performed using Power SYBR Green PCR Master Mix (Vazyme Biotech Co. Ltd) on the Cycler System (Applied Biosystems, USA) according to the manufacturer’s instructions.

We normalized the mRNA content to GAPDH expression within a sample so as to eliminate potential differences in mRNA extraction between samples. Sequences for PCR primers were listed below: NLRP3(F:5′-CAACCTCACGTCACACTGCT-3′, R:5′-TTTCAGACAACCCCAGTTC-3′); ASC(F:5′-CTGACGGATGAGCAGTACCA-3′, R:5′-CAGGATGATTTGGTGGGATT-3′); GAPDH(F: 5′-ATGATTCTACCCACGGCAAG3-′, R: 5′-CTGGAAGATGGTGATGGGTT-3′) Caspase-1(F:5′-ACACGTCTTGCCCTCATTATCT-3′, R:5′-ATAACCTTGGGCTTGTCTTTCA-3′).AIM2(F:5′-TGCTGAATCTGACCAAAAGGT-3′, R:5′- TGTTCCAAGGGGCTGAGTT-3′).The relative level for each mRNA was analyzed using the 2-ΔΔCT method.

### Western blot

Tissues or cells were lysed in RIPA buffer (Sigma) for protein extraction. The protein samples were separated by SDS-PAGE and transferred to polyvinylidene difluoride membranes. Membranes were incubated with primary antibodies (1:1000 or 1:500) against NLRP3 (CST, #15,101), Caspase1 (CST, #24,232), ASC (CST, #67,824), IL-6 (Abcam, ab290750), IL-1β (CST, #27,989), P-P38 (Abcam, ab195049), T-P38 (CST, #8690), P-ERK (Abcam, ab223500), T-ERK (Abcam, ab184699), TLR4 (CST, #14,358), AIM2(CST, #63,660), and GAPDH (Abcam, ab8245) overnight at 4 °C and then for 2 h at room temperature. Finally, the protein signal was quantified using the ImageJ analysis system.

### Cell culture

The H9C2 cell line was cultured in Dulbecco’s modified Eagle’s medium (DMEM) supplemented with 10% fetal bovine serum (FBS), 100 U/mL penicillin, and 100 U/mL streptomycins. The cells were cultured at 37 °C in a humidified 5% CO2 atmosphere. The cells were exposed to PBS, Dox (5 μmol/L), Dox + Dapa (5 μmol/L), and Dox + SB (20 μmol/L) after growing 50–70% of the medium. Following this, the cells were incubated for 24 h with each drug.

### ELISA assays

The supernatant of cells from each group was extracted respectively. According to the guidelines, IL-6, IL-1β, and tumor necrosis factor-α (TNF-α) were detected with a specific ELISA kit (MultiSciences).

### Statistical analysis

A *P* value of < 0.05 was considered statistically significant for all statistical tests, and all tests were two-tailed. GraphPad Prism (version 9.5) was used for all analyses. Graphs depict mean ± SD. One-way ANOVA and Student’s *t*-test were used for comparisons between multiple groups and two groups, respectively. The D'Agostino-Pearson omnibus normality test or Shapiro–Wilk normality test was used to determine whether the data satisfied the condition of normal distribution. *P* < 0.05 was considered significant (*P < 0.05, **P < 0.01, ***P < 0.001, ****P < 0.0001; ns, not significant).

## Results

### Dapagliflozin improved left ventricular function in doxorubicin-induced DCM mice

**Fig. 1:**
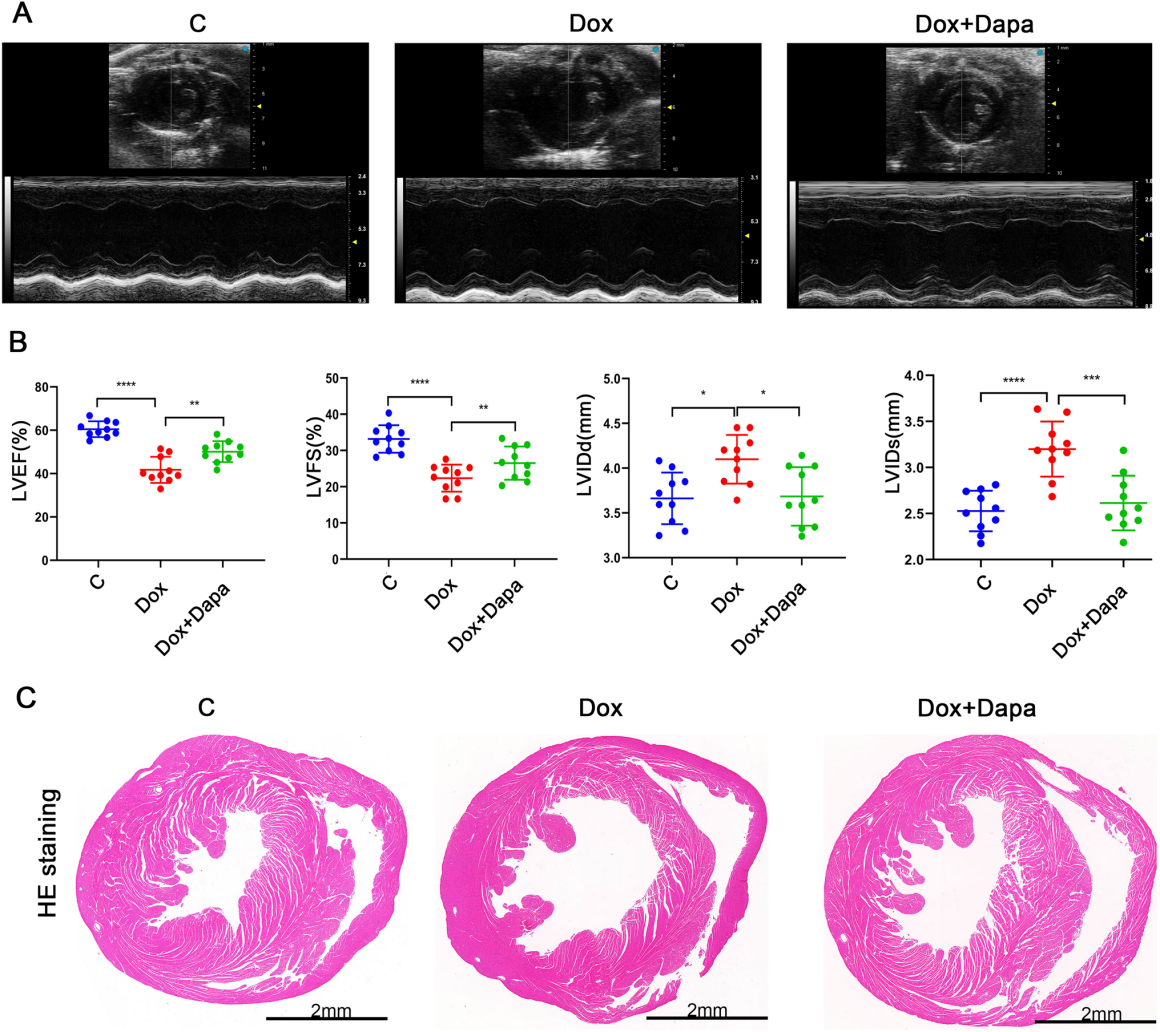
Dapagliflozin treatment significantly improved cardiac functions in doxorubicin-induced DCM mice. **A** Murine cardiac functions are analyzed by echocardiography. **B** The left ventricular ejection fraction (LVEF), the left ventricular fractional shortening (LVFS), the left ventricular internal dimensions (LVIDd), and the left ventricular internal dimensions (LVIDs) were measured at the end of the experiment (*n* = 10 per group). **C** Representative images of the staining of HE in heart sections from mice treated with doxorubicin or dapagliflozin (scale bars, 2 mm)

Echocardiography was used to verify the therapeutic effect of dapagliflozin in mice induced with DCM caused by doxorubicin (Fig. 1A). Mice in the Dox group showed a significant reduction in LVEF (41.75 ± 5.74% versus 60.50 ± 3.44%; *P* < 0.0001) and LVFS (22.36 ± 3.53% versus 33.18 ± 3.59%; *P* < 0.0001), while LVIDd (4.099 ± 0.25 mm versus 3.663 ± 0.27 mm; *P* < 0.05) and LVIDs (3.200 ± 0.28 versus 2.528 ± 0.20 mm; *P* < 0.05) were increased compared to the control group (Fig. 1B). Therefore, the animal model of DCM was successfully constructed. ​Interestingly, treatment with dapagliflozin significantly improved the depressed LVEF (50.11 ± 4.58%versus 41.75 ± 5.74%; *P* < 0.001) and LVFS (26.52 ± 4.33% versus 22.36 ± 3.53%; *P* < 0.0001) and decreased LVIDd (3.684 ± 0.31 mm versus 4.099 ± 0.25 mm; *P* < 0.05) and LVIDs (2.615 ± 0.26 mm versus 3.200 ± 0.28 mm; *P* < 0.005) compared with the Dox group (Fig. 1B). HE-stained hearts were used to examine dapagliflozin’s effect on ventricular dilation in DCM mice (Fig. 1C). According to the results, dapagliflozin reduced doxorubicin-induced dilation of the heart. These results indicate that dapagliflozin prevents cardiac remodeling during the progression of DCM.

### Dapagliflozin inhibited NLRP3 inflammasome activation in doxorubicin-induced DCM mice

**Fig. 2:**
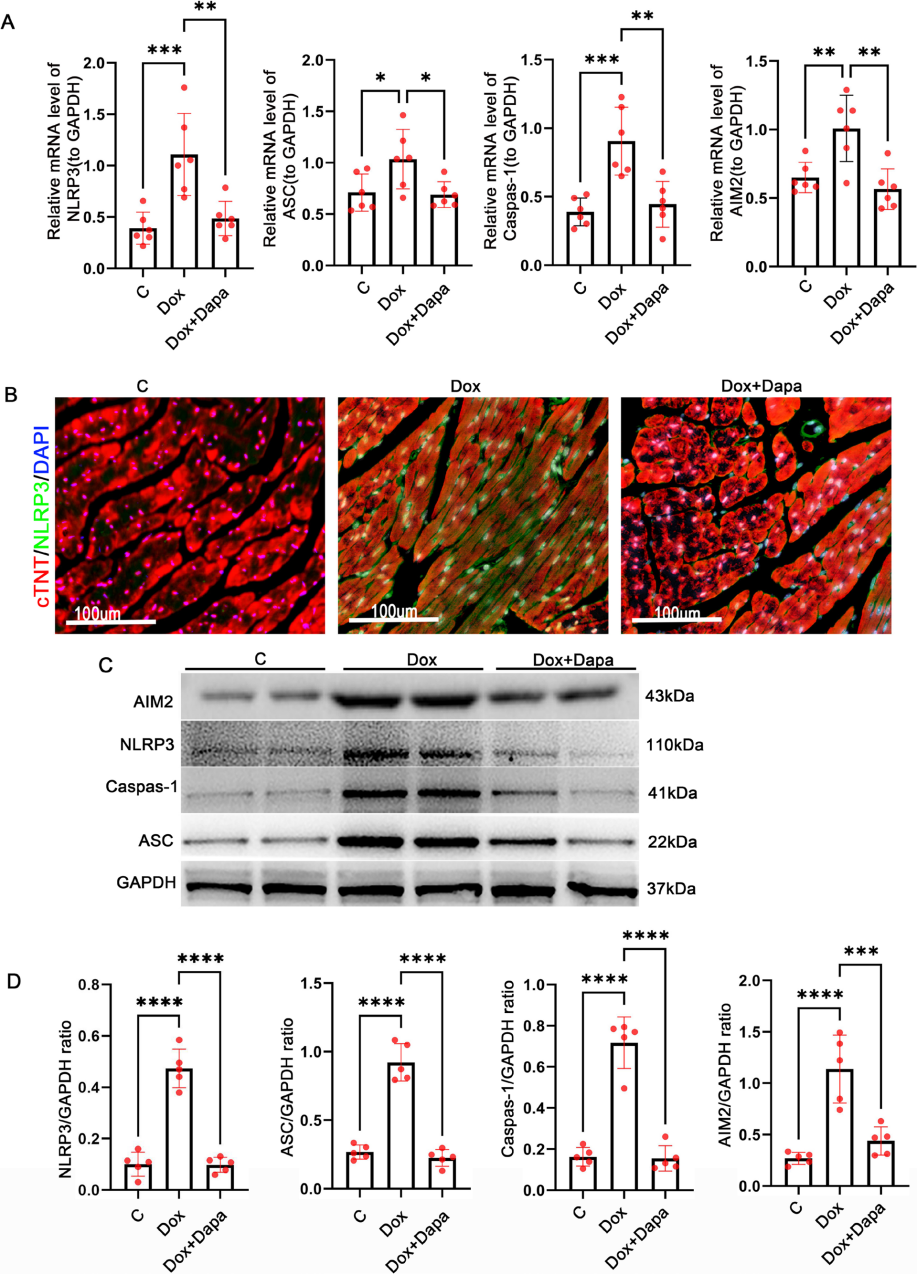
Dapagliflozin inhibited the activation of the NLRP3 inflammasome in doxorubicin-induced DCM mice. **A** Relative NLRP3, ASC, Caspase-1, and AIM2 mRNA expression level were detected by real-time PCR in control, Dox, and Dox + Dapa mice. (*n* = 5 per group). **B** Representative immunofluorescence images of NLRP3 in control, Dox, and Dox + Dapa mice. cTNT (red), NLRP3 (green), and DAPI (blue) (scale bars, 100 μm). **C**–**D** Representative western blot of NLRP3 inflammasome pathway and densitometric analysis in control, Dox, and Dox + Dapa mice (*n* = 5).​ *P < 0.05, **P < 0.01, ***P < 0.001, ****P < 0.0001

Considering that the NLRP3 inflammasome plays a crucial role in the pathophysiology of DCM, we examined the expression of the NLRP3 inflammasome in cardiac tissue. We performed PCR analysis to determine the expression levels of NLRP3 inflammasome-associated genes, including ASC, NLRP3, Caspase-1, and absent in melanoma 2 (AIM2)(Fig. 2A). All of these mRNA expressions were significantly higher in the Dox group than in the control group, while dapagliflozin treatment significantly reduced these mRNA expressions (Fig. 2A) (*P* < 0.05). Consistent with the PCR analysis, immunofluorescent staining indicated that enhanced expression of the NLRP3 inflammasome in the Dox myocardium was also attenuated by dapagliflozin treatment (Fig. 2B). Western blot further confirmed the suppression of dapagliflozin on the upregulated expression of NLRP3 inflammasome (Fig. 2C, D). These findings indicate that dapagliflozin treatment inhibited NLRP3 inflammasome activation in doxorubicin-induced DCM mice.

### Dapagliflozin inhibited the inflammatory factors’ expression in the DCM myocardium tissues

**Fig. 3:**
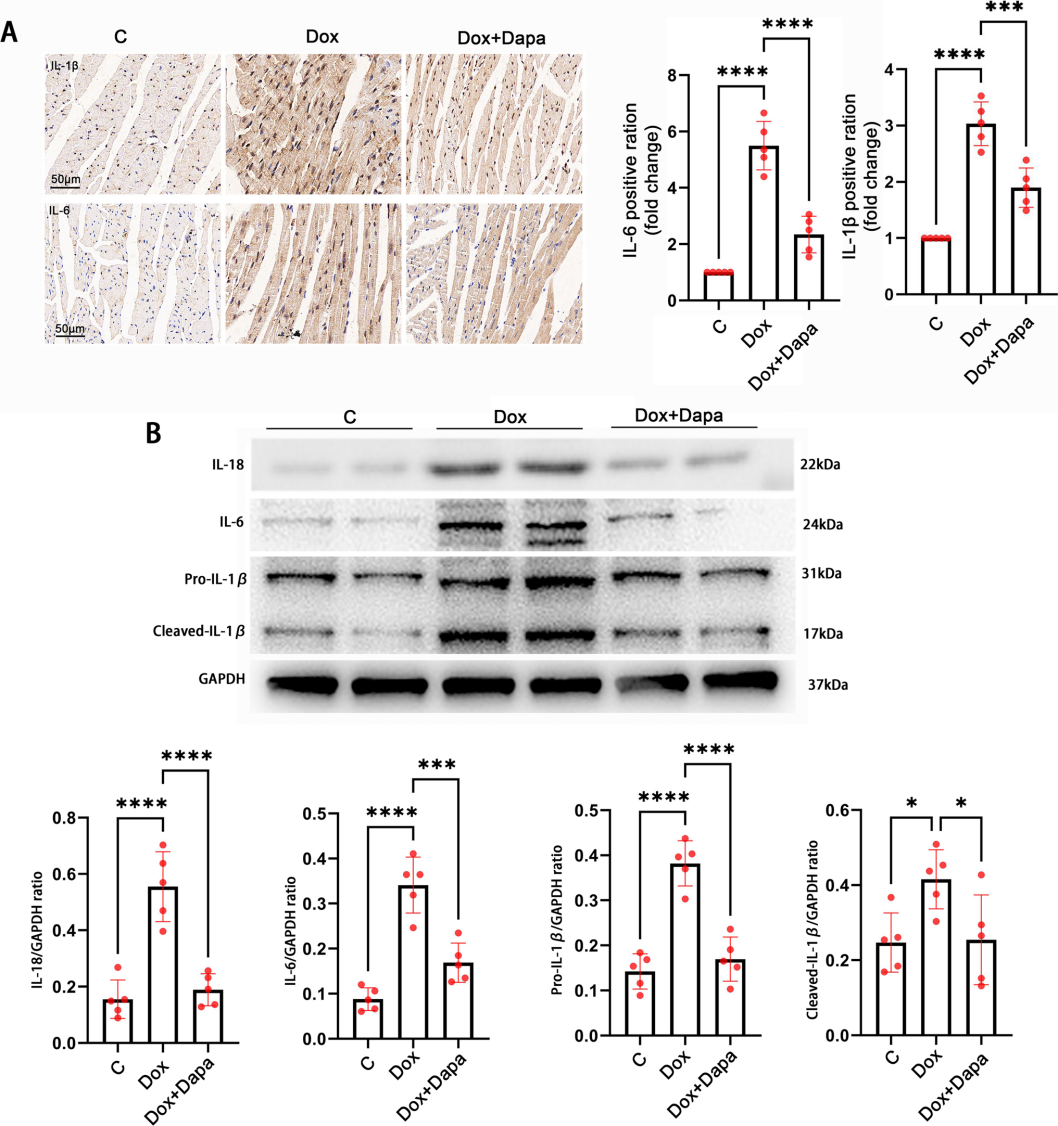
Dapagliflozin inhibited the expression of the inflammatory factors interleukin (IL)-6, L-1β, and IL-18 in cardiac tissues. **A** Representative immunohistochemical images of IL-6, IL-1β, and IL-18 and densitometric analysis in control, Dox, and Dox + Dapa mice (scale bars, 50 μm). **B** Representative western blot of IL-6, pro-IL-1β, cleaved-IL-1β, IL-18, and densitometric analysis in control, Dox, and Dox + Dapa mice (*n* = 5). **P < 0.01, ***P < 0.001, ****P < 0.0001

A pro-inflammatory state is induced when the NLRP3 inflammasome is activated (Broz and Dixit 2016). Therefore, we measured the expression of IL-6, IL-1β, and IL-18 in the myocardium of Dox-induced DCM mice. The immunohistochemical results showed that the expression of IL-6 and IL-1β was significantly downregulated after dapagliflozin treatment compared to the Dox group (Fig. 3A). Consistent with the immunohistochemical results, the western blot showed lower levels of IL-6, IL-1β, and IL-18 in the dapagliflozin treatment myocardium than in the Dox myocardium (Fig. 3B). These results demonstrate that dapagliflozin inhibited the inflammatory factors’ expression in the DCM myocardium tissues.

### Dapagliflozin attenuates the doxorubicin-induced inflammatory response in H9C2 cardiac cells

**Fig. 4:**
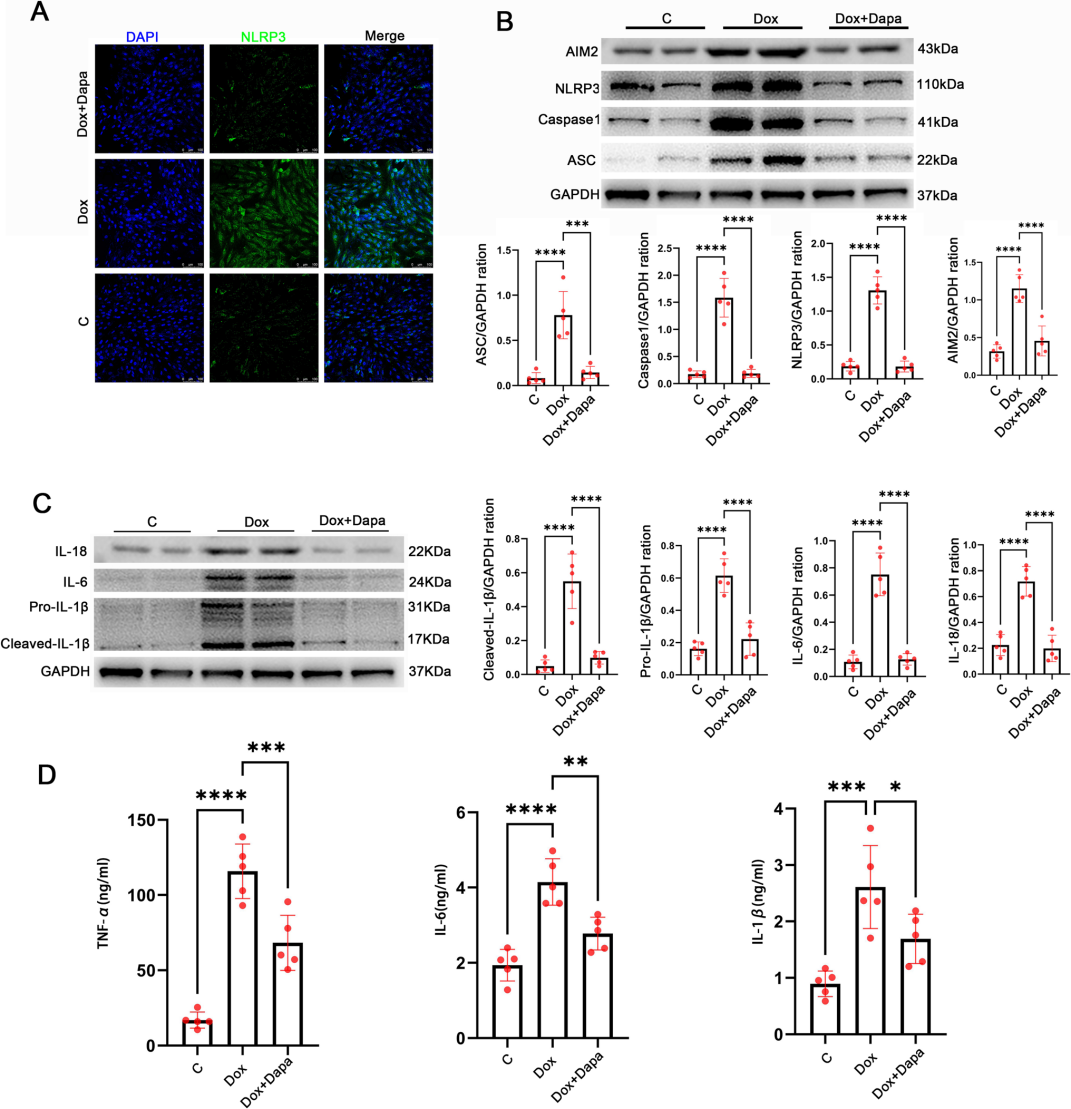
In vitro, dapagliflozin suppressed the Dox-induced inflammatory response. **A** Representative immunofluorescence images of NLRP3 inflammasome in control, Dox, and Dox + Dapa cells (scale bars, 100 μm). **B** Representative western blot of NLRP3 inflammasome pathway and densitometric analysis in control, Dox, and Dox + Dapa cells.​ **C** Representative western blots of inflammatory factors IL-6, pro-IL-1β, cleaved-IL-1β, IL-18, and densitometric analysis in control, Dox, and Dox + Dapa cells. **D** ELISA analysis of inflammatory factors (IL-6, IL-1β, and TNF-α) in cell supernatant of control, Dox, and Dox + Dapa cells. (*n* = 5 per group). ***P < 0.001, ****P < 0.0001

NLRP3 inflammasome activity was detected in H9C2 cardiac cells after treatment with doxorubicin and dapagliflozin in vitro. According to immunofluorescent staining, doxorubicin stimulated the activation of the NLRP3 inflammasome in H9C2 cardiac cells, while dapagliflozin treatment attenuated this effect (Fig. 4A). Additionally, a western blot was performed to detect NLRP3 inflammasome pathway protein levels in H9C2 cardiac cells (Fig. 4B). The results showed that dapagliflozin decreased the activation of the NLRP3 inflammasome pathway in the Dox-treated H9C2 cells. After that, we examined the expression of IL-6, IL-1β, and IL-18 in H9C2 cardiac cells. The results suggested that the expression of IL-6, IL-1β, and IL-18 was significantly downregulated after dapagliflozin treatment compared to the Dox group (Fig. 4C). Besides, the cell supernatant level of IL-6, IL-1β, and TNF-α induced by doxorubicin was significantly reduced by dapagliflozin treatment (Fig. 4D). These results indicate that dapagliflozin reduced the Dox-induced cellular inflammatory response.

### Dapagliflozin reduced the expression of NLRP3 inflammasome through p38-dependent inhibition of TLR4

**Fig. 5:**
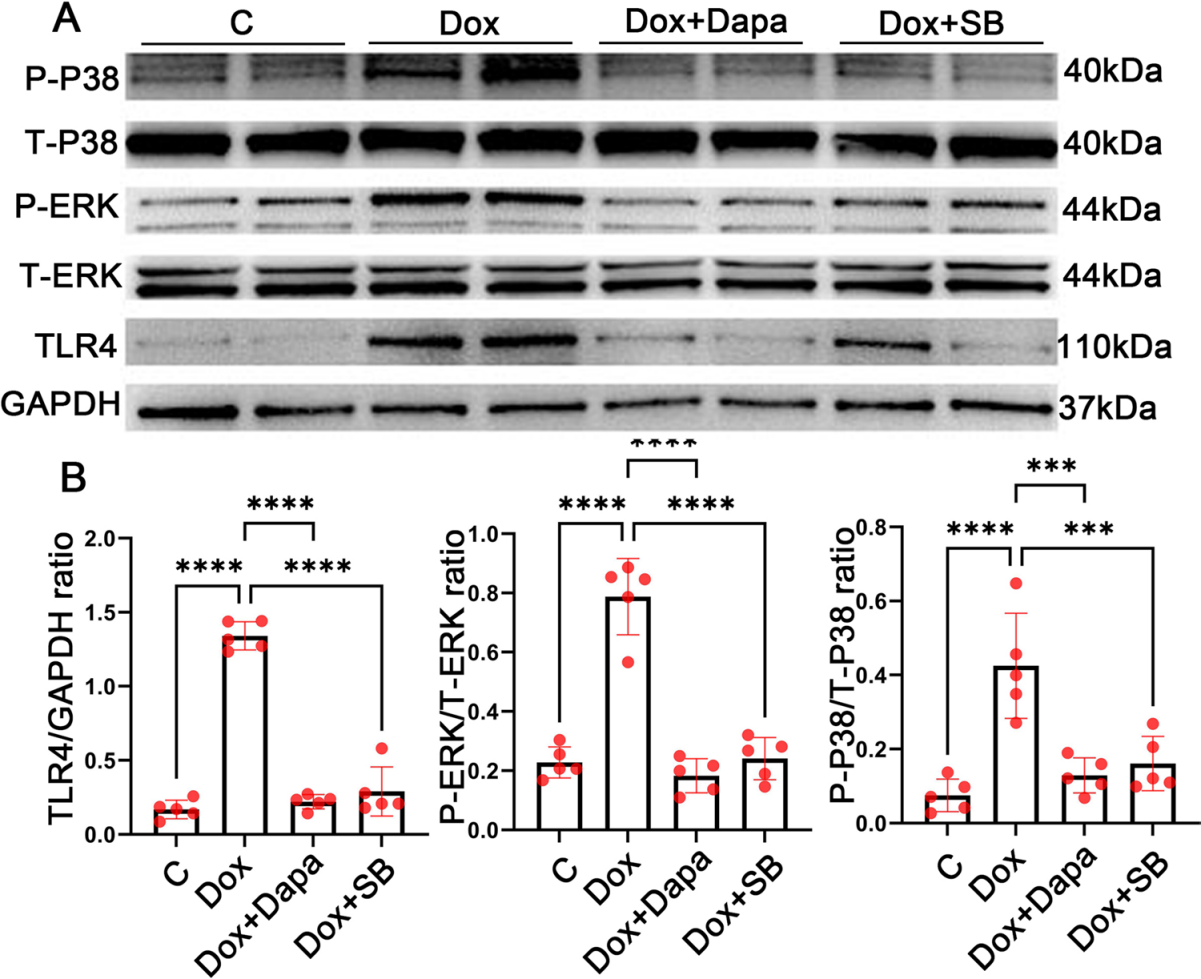
Dapagliflozin reduced the expression of NLRP3 inflammasome through the inhibition of TLR4 by p38. **A**–**B** Representative western blots (**A**) and densitometric analysis (**B**)of TLR4, P-p38, and P-ERK in control, Dox, Dox + Dapa, and Dox + SB cells. ***P < 0.001, ****P < 0.0001

It has been reported that TLR4 expression is increased by p38 phosphorylation, contributing to the activation of NLRP3 (Birnbaum et al. 2019). We wondered if the downregulation of TLR4 was associated with dapagliflozin’s anti-inflammatory effects. The level of protein expression on the P38-ERK-TLR4 pathway is shown in Fig. 5. Dapagliflozin inhibited Dox-induced P-p38, P-ERK, and TLR4 expression. SB203580 (SB), a p38 inhibitor, reduced p38 and ERK phosphorylation and attenuated TLR4 expression, suggesting a p38-dependent effect of dapagliflozin on TLR4 expression. Based on the above results, dapagliflozin suppresses the NLRP3 inflammasome may through p38-dependent downregulation of TLR4.

## Discussion

In this study, a mouse DCM model was used to demonstrate that dapagliflozin prevents ventricular dilatation and improves cardiac function through its anti-inflammatory effects.​Moreover, dapagliflozin treatment also decreased the levels of NLRP3, IL-6, and IL-1β in both DCM myocardium and H9C2 cardiac cells. Furthermore, dapagliflozin also eliminates inflammation by inhibiting NLRP3 inflammasome activation, an effect that is dependent upon p38 activation, which interferes with the downstream attenuation of TLR4 expression.

In general, chronic inflammation plays a crucial role in the pathogenesis of DCM (Maron et al. 2006). ​The NLRP3 inflammasome triggers an inflammatory response by triggering caspase-1, which releases cytokines of the IL-1 family (Toldo et al. 2022; Broz and Dixit 2016).​ In DCM, the NLRP3 inflammasome is activated and mediates pathogenic pyroptosis (Zeng et al. 2020).​ Therefore, targeting NLRP3 inflammasome activation and related pathways may be a potential therapeutic strategy for the treatment of DCM.​ With the activation of the NLRP3 inflammasome, IL-1β and IL-18 are released, which may trigger and amplify inflammation and lead to cardiac injury in DCM (Harding et al. 2022). Doxorubicin also activated the NLRP3 inflammasome in H9C2 cells (Tavakoli Dargani and Singla 2019). Based on our findings, doxorubicin induces inflammasome activation in mouse cardiomyocytes and H9C2 cells in agreement with this view.

Given the significant role of NLRP3 inflammasome in the pathogenesis of DCM, targeted therapeutic agents for NLRP3 inflammasome have received much attention.​ Increasing evidence suggests that SGLT2 inhibitors exert their anti-inflammatory effects through the inhibition of the NLRP3 inflammasome (Takahashi 2022; Ke et al. 2022). In a study in diabetic patients, the SGLT2 inhibitor, empagliflozin, significantly reduced the activity of the NLRP3 inflammasome after 30 days of treatment (Kim et al. 2020). Similarly, we also found that dapagliflozin improved cardiac function in adriamycin-induced DCM mice, suggesting that its anti-inflammatory properties may be clinically applicable.

TLR4 specifically recognizes the LPS of gram-negative bacteria and is involved in various autoimmune diseases (Park and Lee 2013). Studies have shown that TLR4 activation-mediated myocardial leukocyte infiltration results in oxidative stress and the release of cytokines, leading to cardiovascular diseases such as myocarditis, myocardial infarction, and heart failure (Ong et al. 2018; Yang et al. 2016). In recent years, the role of TLR4 activation in the pathogenesis of DCM has been studied. Bangwei Wu et al. reported for the first time that TLR4 activation exacerbates mitochondrial dynamic imbalance and facilitates experimental autoimmune myocarditis (EAM) to DCM progression (Wu et al. 2018). Additionally, Satoh et al. found that TLR4 expression levels in the myocardium were associated with left ventricular dysfunction in human DCM (Satoh et al. 2004). All these results suggest that TLR4 activation may affect the pathophysiological process of DCM. Also, empagliflozin may improve cardiac dysfunction in cardiomyopathy by reducing TLR4 expression in the myocardium (Zhang et al. 2020). Similarly, we also confirmed that dapagliflozin attenuated the increase in P-P38, P-ERK, and TLR4 expression induced by doxorubicin.Further, SB, a p38 inhibitor, also had the same effect as dapagliflozin, suggesting that dapagliflozin may inhibit NLRP3 inflammasome activation by inhibiting the expression of TLR4.

Taken together, these findings suggest that dapagliflozin improves cardiac function in DCM by reducing TLR4 expression and inhibiting NLRP3 inflammasome activation. SGLT2 inhibitors may be a new therapeutic option for patients receiving anthracycline therapy if applied in DCM, as demonstrated in this study.

## Data Availability

Data will be made available on request.
